# ﻿Phylogeography and ecology of bumble bees on Kolguev Island, a remote European Arctic landmass

**DOI:** 10.3897/zookeys.1122.82993

**Published:** 2022-09-20

**Authors:** Grigory S. Potapov, Yulia S. Kolosova, Alexander V. Kondakov, Alena A. Tomilova, Boris Yu. Filippov, Natalia A. Zubrii, Vitaly M. Spitsyn, Elizaveta A. Spitsyna, Alisa A. Zheludkova, Mikhail Yu. Gofarov, Galina V. Bovykina, Ivan N. Bolotov

**Affiliations:** 1 N. Laverov Federal Center for Integrated Arctic Research of the Ural Branch of the Russian Academy of Sciences, Northern Dvina Emb. 23, Arkhangelsk, 163069, Russia N. Laverov Federal Center for Integrated Arctic Research of the Ural Branch of the Russian Academy of Sciences Arkhangelsk Russia

**Keywords:** Bumble bees, High Arctic, island biogeography Pleistocene glaciations, mitochondrial DNA

## Abstract

The bumble bee fauna of the Russian Arctic is rather poorly known. Kolguev Island, a remote insular territory in the Barents Sea, is one of the deficiently studied areas. In this study, material on Kolguev’s bumble bees is re-examined, phylogeographic data analysed, putative scenarios explaining the origin of the bumble bee fauna on the island discussed, and the biology and phenology of these insular populations described. Five bumble bee species, i.e., *Bombusflavidus*, *B.lapponicus*, *B.jonellus*, *B.pyrrhopygus*, and *B.balteatus*, were recorded on this island. All of these species are widespread throughout the Eurasian Arctic. Bumble bee populations on Kolguev Island are characterised by a low level of molecular divergence from mainland populations. Based on paleogeographic reconstructions and phylogeographic patterns, it is hypothesised that the bumble bees appeared on this island in the Early Holocene. The lack of rodents (lemmings and voles) sharply decreases the number of available nesting places for bumble bees on Kolguev Island.

## ﻿Introduction

Kolguev Island is a remote insular territory on the continental shelf in the south-eastern part of the Barents Sea, with a total area of 5130 km^2^ (Lavrinenko and Lavrinenko 2014). This island is composed of Quaternary sediments and is located approximately 70 km north of the coast of Eurasia (Lavrinenko and Lavrinenko 2014). Most of its area is occupied by an accumulative plain, with an average altitude of 20–30 m. However, the southern part of the island is covered by low-elevation wet tundra and peat bogs, while hilly landscapes with average elevations of 80–100 m prevail in its central part.

In the Late Pleistocene, during the period of maximum development of the Scandinavian Ice Sheet, Kolguev Island was a part of the continent due to lower sea levels (Velichko 2002). Available paleogeographic reconstructions reveal that the island area was covered by Arctic deserts (Velichko 2002) or even by massive ice sheets (Svendsen et al. 2004; Hughes et al. 2016). In the Early Holocene, Kolguev Island was isolated from the mainland due to rising sea levels and intense coastal erosion (Velichko 2002).

A review of published literature indicates that the insect fauna of the island may have originated in the Late Pleistocene or Early Holocene (Bolotov 2011). The species diversity of insects on Kolguev Island is rather poorly known, and most available works deal with Lepidoptera and Coleoptera (Buturlin 1903; Semenov 1904; Bolotov 2011; Kullberg et al. 2019; Spitsyn and Bolotov 2020; Potapov et al. 2021b). However, a few publications report on the fauna and species richness of bumble bees collected on Kolguev Island (Berezin 1995a; Kolosova and Potapov 2010, 2011; Potapov et al. 2014; Paukkunen and Kozlov 2020). None of the published entomological works contains data on the phylogeography and biogeographic affinities of insects from Kolguev, including bumble bees.

Bumble bees (genus *Bombus* Latreille) are well adapted to the harsh climatic conditions of the Arctic compared with other groups of bees (Panfilov 1968). The high adaptive capabilities of high-latitude bumble bees could be linked to more effective thermoregulation and shorter life cycle (Berezin 1990, 1995a, b; Radchenko and Pesenko 1994; Goulson 2010). In general, the bumble bee fauna of the Arctic is well studied, especially in Northern Europe and North America (Williams et al. 2014; Rasmont et al. 2021). Conversely, several remote, hard-to-reach areas of the European and Asian Arctic remain poorly studied, including Kolguev Island.

The bumble bee fauna of the Eurasian Arctic can be separated into three distinct groups of species: (1) the High Arctic taxa; (2) the Lower Arctic taxa; and (3) boreal species (Chernov 1978; Chernov and Matveeva 2002). The High Arctic species group contains *Bombushyperboreus* Schönherr, 1809, *B.pyrrhopygus* Friese, 1902, and the polar relict species *B.glacialis* Friese, 1902 (Richards 1973; Chernov and Matveeva 2002; Williams et al. 2019; Potapov et al. 2021a). The latter species is endemic to the Novaya Zemlya Archipelago and Wrangel Island (Potapov et al. 2021a). The Lower Arctic group consists of a number of species such as *B.lapponicus* (Fabricius, 1793), *B.jonellus* (Kirby, 1802), *B.cingulatus* Wahlberg, 1854, and *B.balteatus* Dahlbom, 1832 (Chernov 1978; Chernov and Matveeva 2002). The expansion of boreal bumble bee species associated with species-rich flowering plant associations through river valleys is a common means of enrichment of the Arctic fauna. The boreal species that may colonise the Arctic by this manner are *B.distinguendus* Morawitz, 1869, *B.hortorum* (Linnaeus, 1761), *B.flavidus* Eversmann, 1852, and others (Kolosova and Potapov 2011; Potapov et al. 2019). In general, the species richness of bumble bees in the Arctic and Subarctic regions ranges from two or three on islands to 12–14 at mainland sites (Potapov et al. 2014).

Most of the bumble bee species mentioned above have Palearctic distributions, with the exception of *B.jonellus*, *B.distinguendus*, and *B.flavidus* (Williams 1998). Currently, three Palearctic species of the subgenus Alpinobombus, i.e., B. (A.) pyrrhopygus, B. (A.) balteatus, and B. (A.) hyperboreus, are considered to be distinct species, and are closely related to the Nearctic *B.polaris* Curtis, 1835, *B.kirbiellus* Curtis, 1835, and *B.natvigi* Richards, 1931, respectively (Williams et al. 2015, 2019). However, a number of scholars consider that the Nearctic *B.polaris* and the Palearctic *B.pyrrhopygus* are conspecific and that the older name *B.polaris* should be used for this circumpolar taxon (Rasmont et al. 2021). At the same time, there are different opinions on the distribution of species belonging to the Bombus (Pyrobombus) lapponicus-complex (Sheffield et al. 2020).

This paper aims to (1) re-examine material on Kolguev’s bumble bees using newly collected samples; (2) analyse phylogeographic data and discuss putative scenarios explaining the origin of the bumble bee fauna on Kolguev Island; and (3) describe ecological and phenological patterns for these insular populations.

## ﻿Materials and methods

### ﻿Data sampling, morphological study, and statistical tests

**Figure 1.**
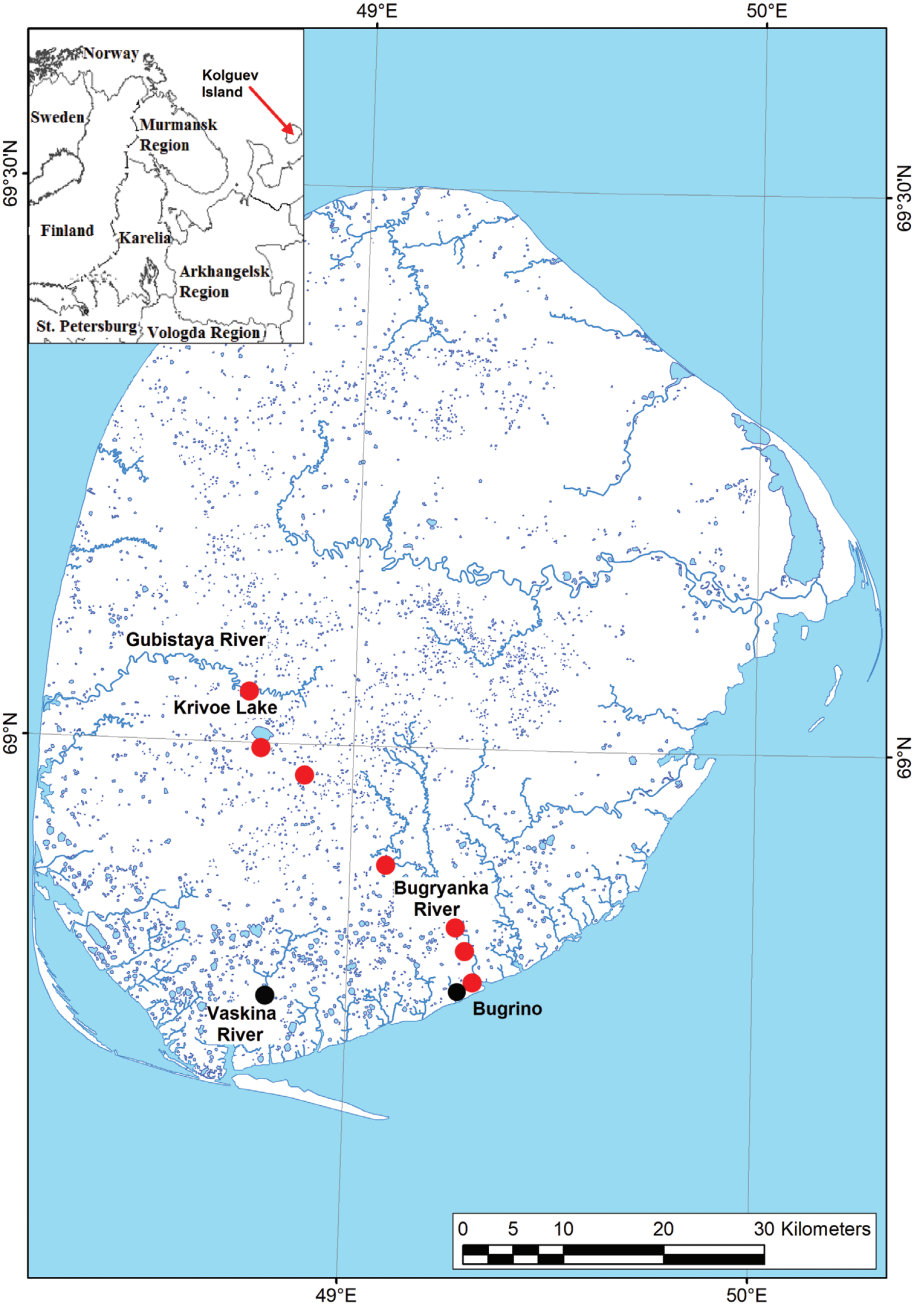
Map of localities on Kolguev Island, from which recent (red circles) and historical (black circles) samples of bumble bees were collected.

Samples of bumble bees from Kolguev Island were collected by Boris Yu. Filippov, Natalia A. Zubrii, Vitaly M. Spitsyn, Alisa A. Zheludkova, Aleksey G. Ardeev, and Grigory S. Potapov in 2009, 2018, and 2020 (total *N* = 287 specimens) (Suppl. material 1: Table S1). The bumble bees were collected in the southern part of the island, i.e., near the village of Bugrino (68.7819°N, 49.3087°E) and along a route from this village to Lake Krivoe (69.0194°N, 48.7211°E) (Fig. 1, Suppl. material 1: Table S1). In most cases, one sample represents a daily sampling effort of a single collector along a walked route of approximately 5 km.

The bumble bees were collected with an entomological net. In some cases, foraging plants of bumble bees were not accurately recorded or bumble bees were caught in flight. For this reason, it is impossible to give a detailed range of the bumble bee foraging resources on Kolguev Island. Our field research was carried out from July to August, allowing us to study phenological patterns for the most abundant species of bumble bees. However, the exact dates of the beginning of the bumble bee flight season on Kolguev Island are unknown because sampling was not possible in May and June.

Specimens of bumble bees from Kolguev Island are deposited in the Russian Museum of the Biodiversity Hotspots (**RMBH**) of the N. Laverov Federal Center for Integrated Arctic Research of the Ural Branch of the Russian Academy of Sciences (Arkhangelsk, Russia). Additional material from the Chukotka and Yamal peninsulas were used for a comparative phylogeographic analysis (Suppl. material 1: Table S1). Only two specimens of bumble bees were found in historical samples from Kolguev Island collected by Buturlin’s (1903) expedition (Suppl. material 1: Table S1). These two specimens were examined in the Zoological Institute of the Russian Academy of Sciences, St. Petersburg (**ZIN**).

Bumble bee specimens were studied using a stereomicroscope Solo 2070 (Carton Optical (Siam) Co., Ltd., Thailand). Bumble bees were identified following Løken (1973, 1984) and Williams et al. (2019). The nomenclature of species follows Williams (1998) and Williams et al. (2019). For the nest description and its measurements, we applied an approach described by Alford (1975) and Martinet et al. (2022).

Statistical procedures (Kolmogorov-Smirnov test for normality and Mann-Whitney test) for the analysis of relative abundance were performed with Statistica v. 13.3 (Stat Soft Inc., USA). We compared the parameters of relative abundance between Kolguev Island and the Novaya Zemlya Archipelago. Novaya Zemlya is the closest insular land to Kolguev Island (approximately 210 km) but differs by having a much larger total area (82,000 km^2^) and harsher environmental conditions (Isachenko 1995).

### ﻿Laboratory protocols

We generated new sequences of the cytochrome c oxidase subunit I (COI) gene from 15 bumble bee specimens (Table 1). The laboratory protocols followed those described in Potapov et al. (2018a, 2018b). Resulting COI gene sequences were checked manually using a sequence alignment editor (BioEdit v. 7.2.5; Hall 1999). The sequencing was carried out at the Engelhardt Institute of Molecular Biology of the Russian Academy of Sciences (Moscow, Russia) using the ABI PRISM BigDye Terminator v. 3.1 reagent kit.

**Table 1.** List of COI sequences for bumble bee specimens used in phylogeographic analyses.

Species	COI haplotype code	COI GenBank/ BOLD IDS acc. no.	Specimen voucher	Locality	References
*B.lapponicus*	LP1	MT053066	RMBH BMB225	Kolguev Island	Potapov et al. (2021a)
*B.lapponicus*	LP1	MT053067	RMBH BMB226	Kolguev Island	Potapov et al. (2021a)
*B.lapponicus*	LP1	MT053068	RMBH BMB227	Kolguev Island	Potapov et al. (2021a)
*B.lapponicus*	LP1	MT053069	RMBH BMB228	Kolguev Island	Potapov et al. (2021a)
*B.lapponicus*	LP1	MT053070	RMBH BMB229	Kolguev Island	Potapov et al. (2021a)
*B.lapponicus*	LP1	OM666877	RMBH BMB102	Yamal: Syoyakha	This study
*B.lapponicus*	LP2	OM666878	RMBH BMB108	Chukotka: 13 km NE from Lorino	This study
*B.lapponicus*	LP3	OM666879	RMBH BMB109	Chukotka: Anadyr	This study
*B.lapponicus*	LP2	OM666880	RMBH BMB111	Chukotka: 13 km NE from Lorino	This study
*B.lapponicus*	LP3	GBHAP756-14	BOMBUS-001	Norway	Gjershaug et al. (2013)
*B.lapponicus*	LP3	GBHAP757-14	BOMBUS-002	Norway	Gjershaug et al. (2013)
*B.lapponicus*	LP3	GBHAP758-14	BOMBUS-006	Norway	Gjershaug et al. (2013)
*B.lapponicus*	LP3	GBHAP759-14	BOMBUS-008	Norway	Gjershaug et al. (2013)
*B.lapponicus*	LP4	GBHAP760-14	BOMBUS-010	Norway	Gjershaug et al. (2013)
*B.lapponicus*	LP3	GBHAP761-14	BOMBUS-014	Norway	Gjershaug et al. (2013)
*B.lapponicus*	LP3	GBHAP762-14	BOMBUS-020	Norway	Gjershaug et al. (2013)
*B.lapponicus*	LP3	GBHAP763-14	BOMBUS-033	Norway	Gjershaug et al. (2013)
*B.pyrrhopygus*	PY1	OM666883	RMBH BMB230	Kolguev Island	This study
*B.pyrrhopygus*	PY10	OM666884	RMBH BMB231	Kolguev Island	This study
*B.pyrrhopygus*	PY10	OM666887	RMBH BMB234	Kolguev Island	This study
*B.pyrrhopygus*	PY1	OM666888	RMBH BMB235	Kolguev Island	This study
*B.pyrrhopygus*	PY1	MK530667	RMBH BMB88	Novaya Zemlya: Malye Karmakuly	Potapov et al. (2019)
*B.pyrrhopygus*	PY1	MK530668	RMBH BMB90	Novaya Zemlya: Malye Karmakuly	Potapov et al. (2019)
*B.pyrrhopygus*	PY1	MK530679	RMBH BMB168	Novaya Zemlya: Bezymyannaya Bay	Potapov et al. (2019)
*B.pyrrhopygus*	PY1	MK530680	RMBH BMB169	Novaya Zemlya: Bezymyannaya Bay	Potapov et al. (2019)
*B.pyrrhopygus*	PY1	MK530681	RMBH BMB170	Novaya Zemlya: Bezymyannaya Bay	Potapov et al. (2019)
*B.pyrrhopygus*	PY1	MK530682	RMBH BMB171	Novaya Zemlya: Bezymyannaya Bay	Potapov et al. (2019)
*B.pyrrhopygus*	PY1	MK530684	RMBH BMB173	Novaya Zemlya: Bezymyannaya Bay	Potapov et al. (2019)
*B.pyrrhopygus*	PY6	OM698596	RMBH BMB199	Chukotka: Anadyr	This study
*B.pyrrhopygus*	PY1	OM698597	RMBH BMB202	Chukotka: Anadyr	This study
*B.pyrrhopygus*	PY1	AF279481	No data	Kamchatka	GenBank
*B.pyrrhopygus*	PY2	KF434342	BOMBUS-029	Norway	Gjershaug et al. (2013)
*B.pyrrhopygus*	PY1	NOAPI563-14	NOAPI563	Norway	BOLD [public record]
*B.pyrrhopygus*	PY3	NOAPI641-14	NOAPI641	Norway	BOLD [public record]
*B.pyrrhopygus*	PY4	WASPS403-14	CCDB-20945 B11	Siberia: Krasnoyarsky Kray	BOLD [public record]
*B.pyrrhopygus*	PY5	WASPS446-14	CCDB-20945 F06	Norway	BOLD [public record]
*B.pyrrhopygus*	PY6	WASPS456-14	CCDB-20945 G04	Norway	BOLD [public record]
*B.pyrrhopygus*	PY7	WASPS466-14	CCDB-20945 H02	Norway	BOLD [public record]
*B.pyrrhopygus*	PY8	WASPS467-14	CCDB-20945 H03	Norway	BOLD [public record]
*B.pyrrhopygus*	PY9	WASPS471-14	CCDB-20945 H07	Norway	BOLD [public record]
*B.balteatus*	BL2	OM666885	RMBH BMB232	Kolguev Island	This study
*B.balteatus*	BL2	OM666886	RMBH BMB233	Kolguev Island	This study
*B.balteatus*	BL3	OM666889	RMBH BMB236	Kolguev Island	This study
*B.balteatus*	BL1	OM666881	RMBH BMB200	Chukotka: Anadyr	This study
*B.balteatus*	BL1	OM666882	RMBH BMB201	Chukotka: Anadyr	This study
*B.balteatus*	BL4	BBWP355-09	1550F10-MON	Mongolia	BOLD [public record]
*B.balteatus*	BL5	NOAPI567-14	NOAPI567	Norway	BOLD [public record]
*B.balteatus*	BL6	WASPS398-14	CCDB-20945 B06	Siberia: Krasnoyarsky Kray	BOLD [public record]
*B.balteatus*	BL7	WASPS399-14	CCDB-20945 B07	Kamchatka	BOLD [public record]
*B.balteatus*	BL1	WASPS423-14	CCDB-20945 D07	Kamchatka	BOLD [public record]

### ﻿Phylogeographic analyses

We used a median-joining network approach using Network v. 5.0.0.1 with default settings (Bandelt et al. 1999). Additional available COI sequences of *B.lapponicus*, *B.pyrrhopygus*, and *B.balteatus* were obtained from the BOLD (the Barcode of Life Data System; Ratnasingham and Hebert 2007) and GenBank databases (*N* = 35; Table 1). Each COI sequence of the aligned datasets was trimmed, leaving a 425-bp fragment for *B.lapponicus*, 455-bp fragment for *B.pyrrhopygus*, and 627-bp fragment for *B.balteatus*. The alignment of COI sequences was performed using the ClustalW algorithm implemented in MEGA7 (Kumar et al. 2016).

## ﻿Results

### ﻿Species richness

Five species of bumble bees were recorded on Kolguev Island, *B.flavidus*, *B.lapponicus*, *B.jonellus*, *B.pyrrhopygus*, and *B.balteatus* (Suppl. material 1: Table S1). During our studies in 2009, 2018, and 2020, *B.pyrrhopygus* and *B.lapponicus* were the most common and widespread taxa on the island (*N* = 124 and 123 specimens, respectively), while *B.balteatus* was observed less frequently (*N* = 43 specimens). Six specimens of *B.flavidus* were collected on this island and only one specimen of *B.jonellus* was sampled in 2009.

Relative abundance of bumble bees on Kolguev Island is 6.27±1.05 specimens per sample (mean ± s.e.; *N* = 48; in most cases, one sample represents a daily sampling effort of a single collector along a walked route of approximately 5 km), which is two times higher than that on Novaya Zemlya, with 3.11±0.46 specimens per sample (mean ± s.e.; *N* = 44; data from Potapov et al. 2019: table 1). The differences between these insular areas in relation to their relative bumble bee abundances are highly significant (Mann-Whitney test: *U* = 711, *P* = 0.0058). The mean number of recorded species per sample (± s.e.) on Kolguev Island and Novaya Zemlya is 1.94±0.13 (*N* = 48) and 1.43±0.10 (*N* = 44), respectively. This parameter is also higher on the first island (Mann-Whitney test: *U* = 707, *P* = 0.0029).

### ﻿Phylogeography

**Figure 2.**
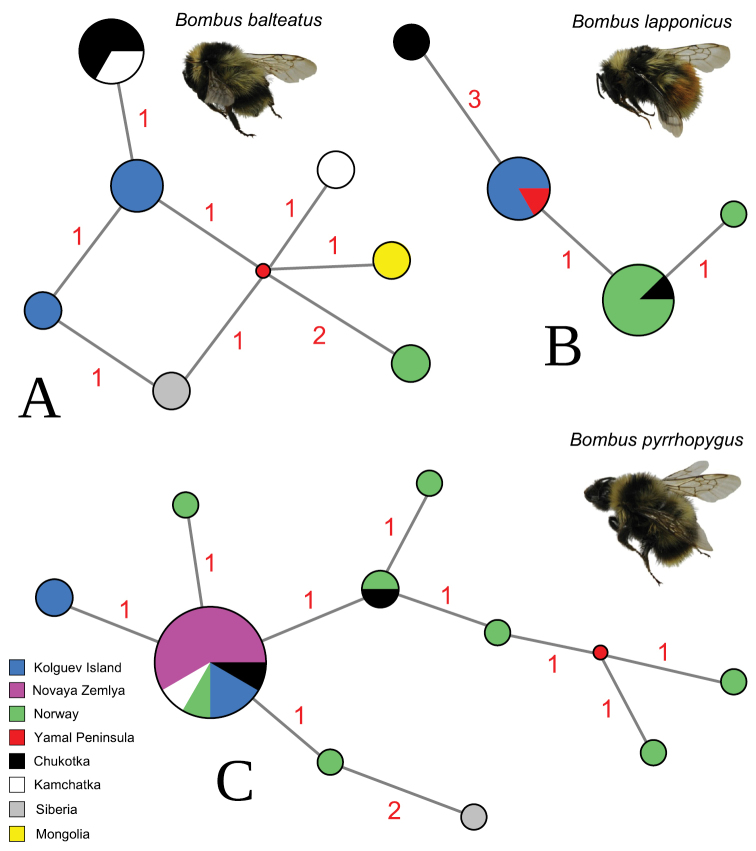
Median-joining haplotype networks of the available COI sequences of widespread bumble bees from Kolguev Island and other Arctic areas **A***Bombusbalteatus***B***B.lapponicus***C***B.pyrrhopygus*. The circle size is proportional to the number of available sequences belonging to a certain haplotype (smallest circle = one sequence). The small red dots indicate hypothetical ancestral haplotypes. Red numbers near branches indicate the number of nucleotide substitutions between haplotypes.

We found that the sequenced *B.lapponicus* specimens from Kolguev Island belong to a single COI lineage (haplotype LP-1) that also occurs in the population from Yamal (Table 1, Fig. 2). *Bombuspyrrhopygus* shares two COI haplotypes on Kolguev, one of which is also known to occur in Norway, Novaya Zemlya, Chukotka, and Kamchatka (haplotype PY1). In contrast, the second haplotype of this species (PY10) was not recorded anywhere else but is genetically close to the first lineage. *Bombusbalteatus* from Kolguev Island also shares two COI haplotypes (BL2 and BL3), which were not recorded anywhere else (Table 1, Fig. 2).

### ﻿Colour variations

*Bombuspyrrhopygus* and *B.balteatus* are highly variable in their colour patterns (Løken 1973; Williams et al. 2019; Rasmont et al. 2021). The dark form of *B.pyrrhopygus*, which is known to occur in Scandinavia and the Kola Peninsula, was not recorded on Kolguev Island. Specimens of this species collected on Kolguev Island share a colour variation of tergites T4–T6. It ranges from a black coloration without ferruginous to a quite distinct ferruginous colouration. Regarding *B.balteatus*, the colour variation of T4–T6 from yellowish white to whitish occur in a series of specimens from Kolguev Island.

### ﻿Bumble bee habitats, foraging resources, and phenological patterns

**Figure 3.**
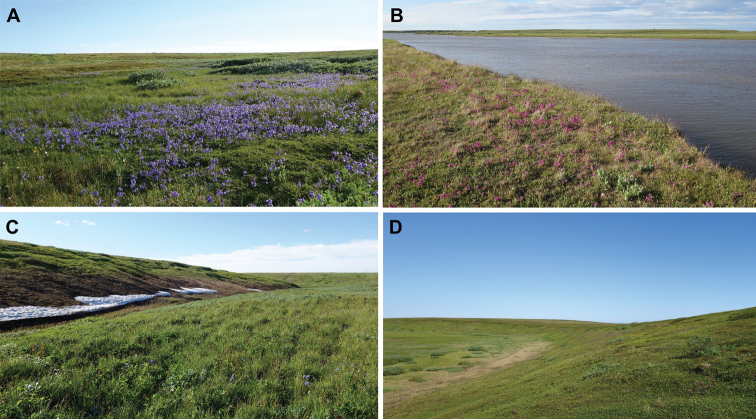
Habitats and foraging resources of bumble bees on Kolguev Island **A** willow-sedge tundra with *Polemoniumacutiflorum*, 10.vii.2020 **B** meadow-like associations with *Pedicularis* sp., shore of the Bugryanka River, 10.vii.2020 **C** willow-sedge tundra with *Geumrivale* and *Polemoniumacutiflorum* along a stream valley, 11.vii.2020 **D** willow-grass tundra on slopes with *Pedicularis* sp., 18.vii.2020.

The main places of aggregation of foraging bumble bee individuals in the southern part of Kolguev Island are river valleys, where foraging resources and appropriate nesting places are concentrated (Fig. 3). Bumble bees rarely occur beyond these areas because continuous wet tundra and peat bog landscapes between river valleys are unfavourable for foraging and the establishment of colonies. Different species of bumble bees do not vary in their habitat preferences on Kolguev Island.

Bumble bees have been recorded on water avens (*Geumrivale*), whorled lousewort (*Pedicularisverticillata*), hairy lousewort (*Pedicularishirsuta*), tall Jacob’s ladder (*Polemoniumacutiflorum*), candle spur (Delphiniumelatumvar.hirsutum), cloudberry (*Rubuschamaemorus*), and on different willow species (*Salix* spp.).

**Figure 4.**
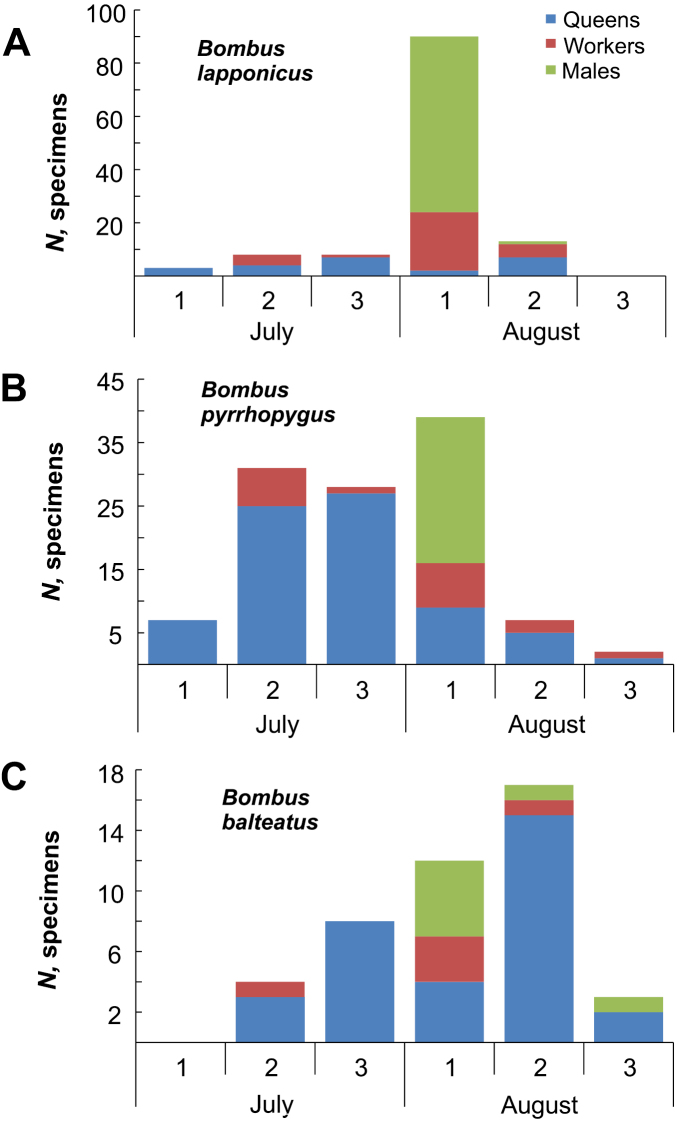
Phenology of bumble bees from Kolguev Island by ten-day periods **A***Bombuslapponicus* (*N* = 122 specimens) **B***B.pyrrhopygus* (*N* = 114 specimens) **C***B.balteatus* (*N* = 44 specimens).

Queens of bumble bees were recorded from early July to late August (Fig. 4). The majority of workers were collected in late July and early August, whereas males were caught in August. The final date when a bumble bee (*B.pyrrhopygus*) was recorded on Kolguev Island was 30 August (Suppl. material 1: Table S1).

### ﻿Nest of *B.lapponicus*

**Figure 5.**
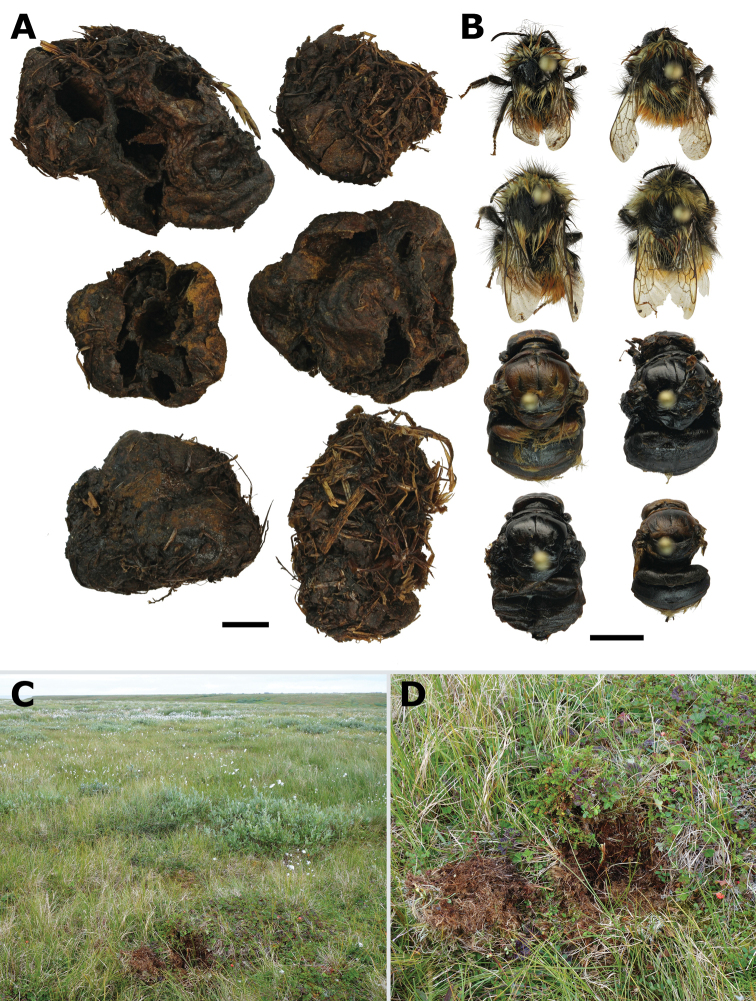
The nest of *Bombuslapponicus* on Kolguev Island **A** excavated nest **B** workers were found alive in the nest (four upper specimens), and those collected dead from cocoons (four lower specimens) **C** nesting site in tundra with cottongrass, crowberry, cloudberry, dwarf birch, and willows **D** tussock with the excavated nest. Scale bars: 5 mm.

One nest of *B.lapponicus* was found on Kolguev Island (15 August 2018, Bugryanka River valley, 68.802861°N, 49.299528°E, Potapov and Zheludkova leg.) (Fig. 5). This nest was located in a tundra site, the plant cover of which was dominated by cottongrass (*Eriophorum* sp.), crowberry (*Empetrumnigrum*), cloudberry (*Rubuschamaemorus*), dwarf birch (*Betulanana*), and willows (*Salix* spp.). The nest was situated inside a tussock and contained 24 cocoons, of which 20 were empty. The mean size (± s.e.) of the measured cocoons is as follows: length 13±0.3 mm, width 10±0.2 mm (*N* = 8). The size of cocoons from the initial pupal clump is as follows: length 8±0.1 mm, width 7±0.1 mm (mean ± s.e.; *N* = 4). There were four living workers of *B.lapponicus* inside the nest, while four other workers were taken dead from cocoons. The length of living workers was 12, 11, 9, and 8 mm; the dead workers from cocoons were 13, 12, 12, and 8 mm long.

## ﻿Discussion

### ﻿Bumble bee fauna of Kolguev Island

The fauna of bumble bees on Kolguev Island with five species (*B.flavidus*, *B.lapponicus*, *B.jonellus*, *B.pyrrhopygus*, and *B.balteatus*) is similar to other species-poor Arctic faunas, dominated by cold-adapted bumble bee species (Potapov et al. 2019). The bumble bee fauna of Vaygach Island (the total area 3400 km^2^, geographic distance approximately 340 km from Kolguev) is the most similar to the studied fauna. In particular, five bumble bee species occur on Vaygach Island, i.e., *B.flavidus*, *B.lapponicus*, *B.pyrrhopygus*, *B.balteatus*, and *B.hyperboreus* (see Potapov et al. 2017).

We have no reliable records of *B.hyperboreus* from Kolguev Island because earlier references to the existence of this species on the island (Kolosova and Potapov 2010, 2011; Rasmont et al. 2021) were incorrect. However, future records of *B.hyperboreus* on Kolguev Island can be expected, mainly in the central and northern part of this island, where the species richness of insects (e.g., Lepidoptera: Bolotov 2011) is higher due to the prevalence of hilly landscapes with richer plant diversity (Potapov et al. 2021b).

Only one specimen (queen) of *B.jonellus* was found on Kolguev Island in 2009. However, in subsequent studies, this species was not rediscovered there. As in the case of *B.hyperboreus*, additional research is needed in the central and northern parts of Kolguev Island. It is also possible that our solitary record of *B.jonellus* reflects an unsuccessful colonisation event because no workers and males of this species were recorded on Kolguev Island. It is well known that queens of bumble bees may migrate for quite considerable distances and that they are not deterred by larger water barriers (Fijen 2020). In the case of Kolguev Island, located 70 km from the mainland, the possibility of an accidental dispersal of a *B.jonellus* female to the island cannot be excluded. Earlier, two migrant butterfly species were recorded on Kolguev Island (Bolotov et al. 2021).

A few specimens of *B.flavidus* were recorded on Kolguev Island, i.e., one female and five males. This cuckoo bumble bee is known as a social parasite of *B.lapponicus* (see Lhomme and Hines 2019), which is a common and widespread species on the island. Earlier reference to the record of *B.norvegicus*, another cuckoo bumble bee species, on Kolguev Island (Kolosova and Potapov 2010, 2011; Rasmont et al. 2021) is incorrect. It was based on a misidentification of a *B.flavidus* female. Hence, Kolguev’s fauna contains only one species of cuckoo bumble bee, *B.flavidus*.

Finally, records of *B.glacialis* Friese, 1902 on Kolguev Island, mentioned by earlier scholars (Pittioni 1943), were not confirmed by our recent surveys (Potapov et al. 2021a). The latter species is a Pleistocene glacial relict and is endemic to Novaya Zemlya and Wrangel Island (Potapov et al. 2018a, 2019, 2021a). It is unlikely that *B.glacialis* is present on Kolguev Island due to the significant environmental differences between Kolguev and Novaya Zemlya.

### ﻿Phylogeographic pattern in the populations of Kolguev’s bumble bees and a prospective scenario of their expansion to this island

We analysed the COI sequences of three widespread species of bumble bees on Kolguev Island and found that they belong to common Northern Eurasian lineages. Kolguev’s *B.lapponicus* reveals a single COI haplotype that also occurs in a population from Yamal and is close to the Norwegian lineage. *Bombuspyrrhopygus* shares two haplotypes, which are also known to occur in Norway, Novaya Zemlya, Chukotka, and Kamchatka. *Bombusbalteatus* from Kolguev Island have two haplotypes, which were not recorded anywhere yet, but they are close to the COI lineage from Chukotka, Kamchatka, and Siberia. In summary, all three species from Kolguev share a low level of molecular divergence from mainland populations, which aligns with the results of earlier phylogeographic research on *B.hyperboreus* and *B.pyrrhopygus* from Novaya Zemlya (Potapov et al. 2019).

We hypothesise that *B.lapponicus*, *B.pyrrhopygus*, and *B.balteatus* spread across the emerged Eurasian shelf margin in the Late Pleistocene, with subsequent fragmentation of their continuous ranges in the Holocene. Taking into account the geological history of the region (Velichko 2002) and our data on bumble bee phylogeography, we conclude that the bumble bees appeared on Kolguev Island no earlier than the Early Holocene, as did some other animal species such as a tiger moth (Bolotov et al. 2015) and a freshwater fish (Artamonova et al. 2020). During the Last Glacial Maximum, Kolguev Island was covered by Arctic deserts or the ice sheet. After the Holocene Climate Optimum, the vegetation cover on the island shifted to tundra ecosystems (Velichko 2002), which are more suitable as habitats for cold-tolerant Arctic bumble bees.

### ﻿The life cycle and ecology of the Kolguev’s bumble bees

The three most common species of the insular fauna (*B.lapponicus*, *B.pyrrhopygus*, and *B.balteatus*) are widespread throughout Kolguev Island but *B.balteatus* occurs less frequently. Obviously, the flight activity of bumble bees is dependent on weather conditions. Their flight season is typical for the Arctic territories with the maximum abundance of individuals in the warmest period. On Kolguev Island this period lasts from the second half of July to the first half of August and is characterised by a mean air temperature of 8 °C (Potapov et al. 2019, 2021a). We have no exact dates of the earliest emergence of bumble bee queens on Kolguev Island. As in the case of other Arctic islands, it should be sometime between mid-May and mid-June (Potapov et al. 2019, 2021a).

No bumble bee nests have been recorded on Kolguev Island prior to our recent discovery of a nest of *B.lapponicus*, described herein. This nest was found in mid-August, when the life cycle of *B.lapponicus* on Kolguev Island enters its final stage. Hence, we did not have the opportunity to examine several aspects of the species’ development such as the emergence of the first-brood adults, behaviour of workers in the nest, and the emergence of males. From available data, we can only conclude that the nest on Kolguev is typical for this species (Martinet et al. 2022). The number of individuals in the colony was quite small, which is typical for bumble bee colonies from Arctic territories (Berezin 1990).

The complete absence of rodents (e.g., lemmings and voles) is a unique feature of Kolguev Island that influences the animal life of the island in several ways, especially by switching the Arctic predators from rodents to other prey resources (Pokrovsky et al. 2015). It is unknown how exactly the absence of lemmings affects the bumble bees of Kolguev Island, but in the Arctic, bumble bees frequently use lemming burrows as nesting sites. Hence, the abundance of bumble bees increases in areas with higher concentrations of lemmings and their burrows (Berezin 1990, 1995b; Potapov et al. 2021a). The lack of lemming burrows considerably limits the nesting places of bumble bees on Kolguev Island. It seems that bumble bees use every available resource such as tussocks, edges of river terraces, and human buildings to establish a nesting colony on the island.

The abundance of bumble bees on Kolguev Island is rather low but the mean value (number of specimens per sampling effort) is two times higher than that on Novaya Zemlya (Potapov et al. 2019, 2021a). The total and mean species richness of bumble bees on Kolguev Island is also higher compared with those on Novaya Zemlya. These differences could be explained by specific landscape and climatic features of Kolguev Island, which is a plain insular landmass, taking a more southern geographic position compared with that of the mountainous Novaya Zemlya Archipelago (Potapov et al. 2019, 2021a). However, a possible role of the interannual variability in weather conditions (sampling in the two areas was made in different years) may also be considered there.

## Supplementary materials

Supplementary material 1 Table S1 Data type Specimen data Explanation note Specimens of bumble bees examined under this study. https://binary.pensoft.net/file/746103This dataset is made available under the Open Database License (http://opendatacommons.org/licenses/odbl/1.0/). The Open Database License (ODbL) is a license agreement intended to allow users to freely share, modify, and use this Dataset while maintaining this same freedom for others, provided that the original source and author(s) are credited.Grigory S. Potapov, Yulia S. Kolosova, Alexander V. Kondakov, Alena A. Tomilova, Boris Yu. Filippov, Natalia A. Zubrii, Vitaly M. Spitsyn, Elizaveta A. Spitsyna, Alisa A. Zheludkova, Mikhail Yu. Gofarov, Galina V. Bovykina, Ivan N. Bolotov
